# Computed tomography angiography-based analysis of high-risk intracerebral haemorrhage patients by employing a mathematical model

**DOI:** 10.1186/s12859-019-2741-5

**Published:** 2019-05-01

**Authors:** Le Zhang, Jin Li, Kaikai Yin, Zhouyang Jiang, Tingting Li, Rong Hu, Zheng Yu, Hua Feng, Yujie Chen

**Affiliations:** 1 College of Computer and Information Science, Southwest University, Chongqing, 400715 People’s Republic of China; 2 College of Computer Science, Sichuan University, Chengdu, 610065 People’s Republic of China; 3 Medical Big Data Center, Sichuan University, Chengdu, 610065 People’s Republic of China; 4 School of Medical Information and Engineering, Southwest Medical University, Luzhou, 646000 People’s Republic of China; 5 Department of Neurosurgery, Southwest Hospital, Third Military Medical University, Chongqing, 400038 People’s Republic of China; 6 School of Mathematics and Statistics, Southwest University, Chongqing, 400715 People’s Republic of China; 7 Department of Neurosurgery, Fuling Central Hospital, Chongqing, 400715 People’s Republic of China

**Keywords:** Intracerebral haemorrhage, Computed tomography angiography, Ensemble learning, Lenticulostriate arterial, data mining

## Abstract

**Background:**

Haemorrhagic stroke accounts for approximately 31.52% of all stroke cases, and the most common origin is hypertension. However, little is known about the method to identify high-risk populations of hypertensive intracerebral haemorrhage.

**Results:**

The results showed that the angle between the middle cerebral artery and the internal carotid artery (AMIC), the distance between the beginning of the median artery and superior trunk (DMS), and the density (CT value) of the lenticulostriate artery (CTL) were statistically significant enough to cause intracerebral haemorrhage. In addition, we chose these three potential features for the ensemble learning classification model. Our developed ensemble-learning method outperforms not only previous work but also three other classic classification methods based on accuracy measurements.

**Conclusions:**

The developed mathematical model in the present study is efficient in predicting the probability of intracerebral haemorrhage.

**Electronic supplementary material:**

The online version of this article (10.1186/s12859-019-2741-5) contains supplementary material, which is available to authorized users.

## Background

Haemorrhagic stroke accounts for approximately 31.52% of all strokes, and the most common origin is hypertension [1]. The most frequent location of hypertensive intracerebral haemorrhage is around the basal ganglia and thalamus, which could easily lead to death or disability [2]. The most prevalent risk factors among stroke survivors are hypertension (88%), smoking (48%), and alcohol use (44%) [3]. However, we know little about the method to identify high-risk populations of hypertensive intracerebral haemorrhage. Since the pre-diagnosis of intracerebral haemorrhage can effectively reduce the incidence rate of intracerebral haemorrhage [4–8], this research develops a mathematical model for the prediction of the probability of intracerebral haemorrhage.

Recently, computational biologists employed information technologies to predict haematoma expansion after intracerebral haemorrhage and the prognosis of intracerebral haemorrhage [9–11], but only a few studies consider the haemorrhage risk for the non-intracerebral haemorrhage patients. Previous studies [12, 13] already used external factors (i.e., temperature and weather [12, 13]) as the features to build up the predictive models for intracerebral haemorrhage. However, these models can only predict population occurrence probability for the intracerebral haemorrhage, and it is difficult for us to predict the probability of intracerebral haemorrhage and obtain high predictive accuracy for each patient.

Usually, we employ computed tomography (CT) images to predict the haematoma enlargement after intracerebral haemorrhage, or the historical characteristics and biomarkers for the neurological outcome prediction of intracerebral haemorrhage patients. Recently, computational biologists started using density, area and other factors of the abnormal area of haematoma in CT images to develop a predictive model for haematoma enlargement after intracerebral haemorrhage [14, 15]. However, since recent studies could not collect adequate training and testing data, they only predicted the degree of deterioration of intracerebral haemorrhage but could not effectively estimate the probability of intracerebral haemorrhage for these patients.

To overcome the shortcomings for previous predictive models [5–8, 10, 11], this study proposes a mathematical framework for the prediction of intracerebral haemorrhage with the following three innovations. First, statistical methods are employed to compute the size of the samples and demonstrate which candidate biomarkers are statistically significant enough to be the features of the model. Second, the predictive model is built with these features. Finally, a novel ensemble-learning method [16] is developed by integrating the support vector machine (SVM) [17], decision tree [18] and K-Nearest Neighbor(KNN) [19] algorithms into the model to improve the predictive accuracy.

Our research results reveal that AMIC, DMS and CTL can predict intracerebral haemorrhage. Moreover, we use them to build the ensemble-learning predictive model, which not only outperforms the classic SVM, decision tree and KNN models but also performs better than previous similar research [20–22].

## Methods

### Experimental materials

A total of 151 patients with unilateral hypertensive ICH admitted to the Department of Neurosurgery, Southwest Hospital, Chongqing, China and confirmed with computed tomography angiography between January 2012 and December 2016 were analysed retrospectively. The protocol was approved by the Ethics Committee of Southwest Hospital, and the committee waived the need for patient consent due to the retrospective nature of the study. The basic information of these patients is listed in Additional file 1: Tables S1 and Table S2. All patients’ computed tomography angiography images were analysed from the haematoma side and non-haematoma side as a self-controlled study of the high-risk features of intracerebral haemorrhage.

**Fig. 1:**
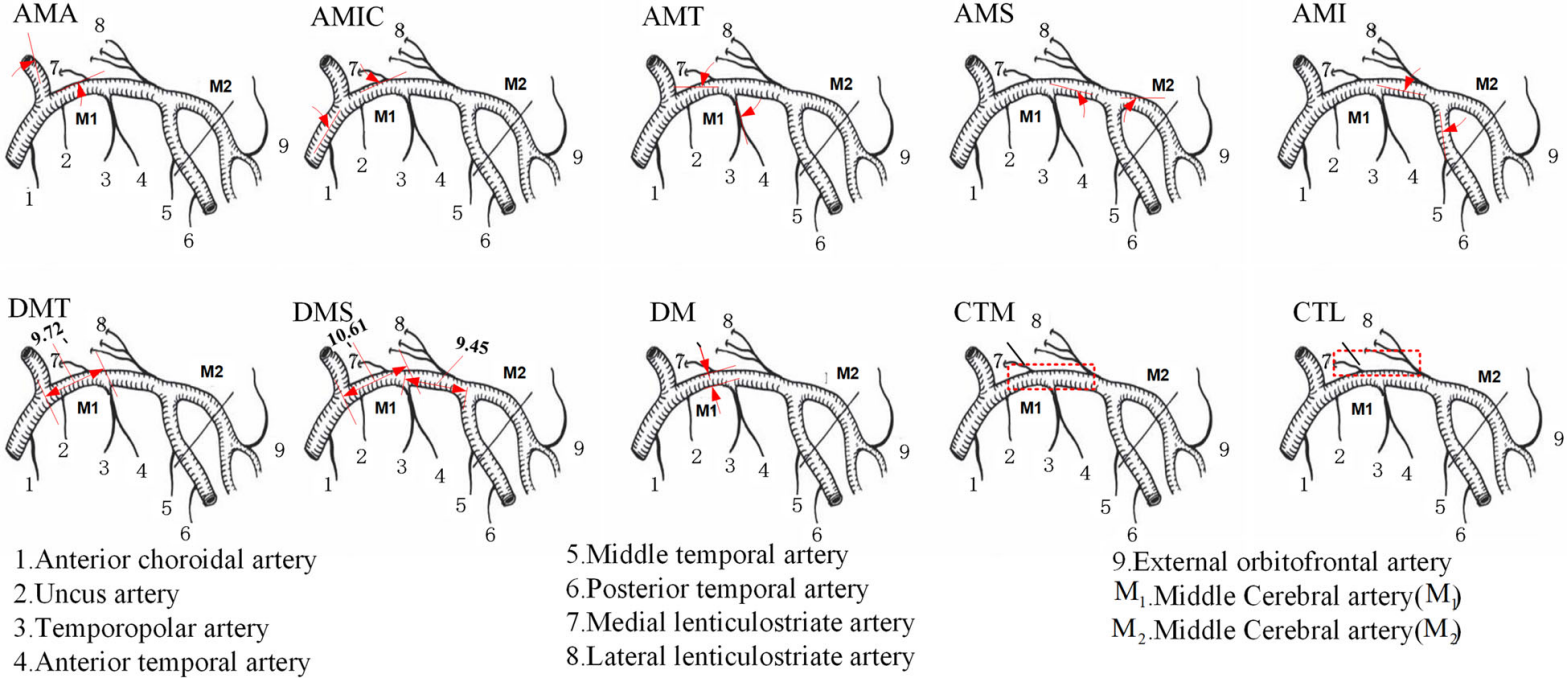
Images of ten potential features

Since haemodynamics around the middle cerebral artery and lenticulostriate artery are tightly associated with the blood vessel rupture for intracerebral haemorrhagic patients [23–25], we selected ten potential features (Fig. 1) related to the artery angle, artery distance, and artery density as the candidate features for model development in this area (Additional file 1: Table S3).

This study recruited 151 CT angiography imaging series, and we used RadidaDicomViewer software [26] to extract the digital values of the potential features (Additional file 1: Table S4) from the samples.

### Model development

**Fig. 2:**
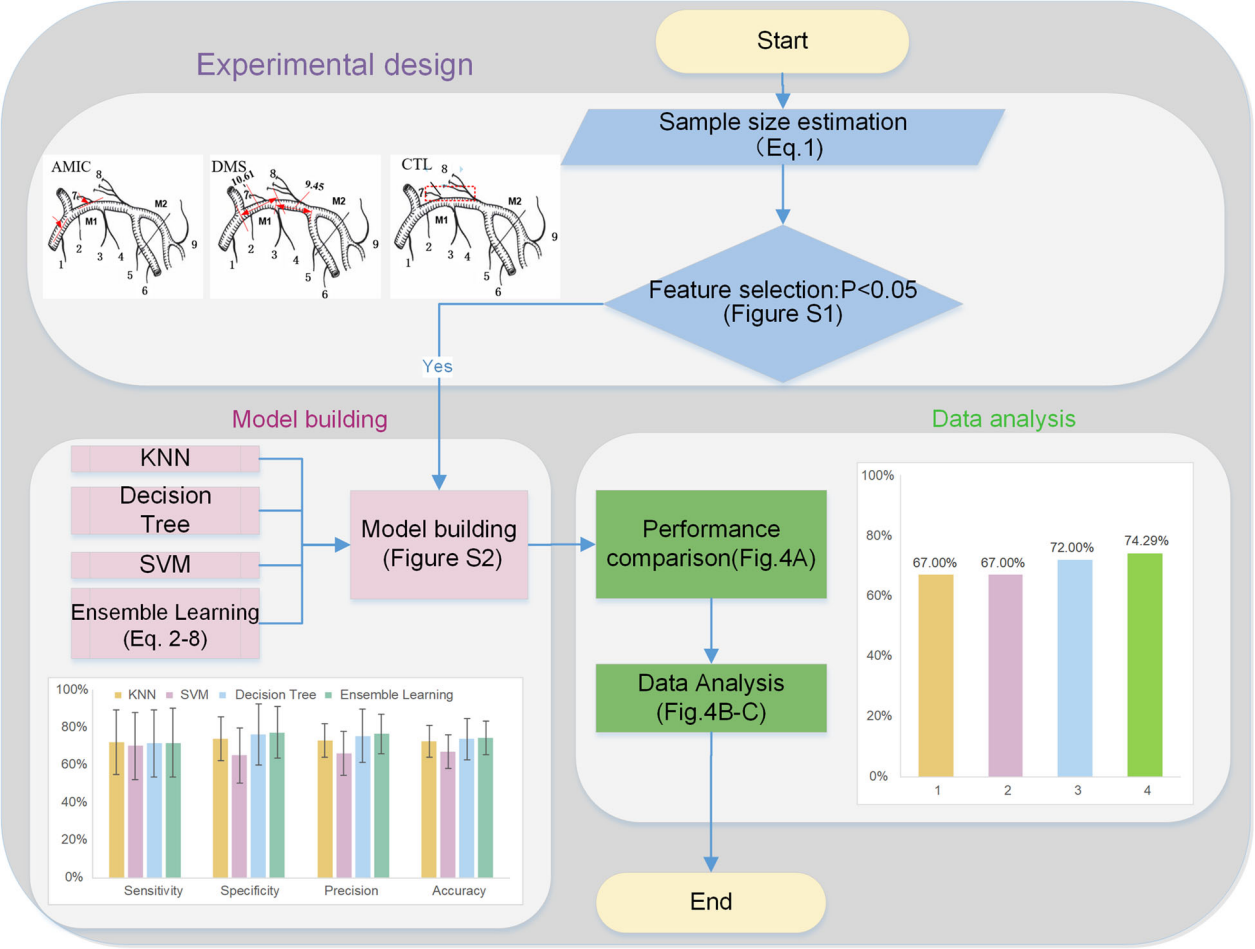
Workflow of the research

Figure 2 shows the workflow of the mathematical model. The first step of the study was using the experimental design to determine the size of the sample and employing statistical tests to choose the candidate features for the model from the potential features. Second, we developed a novel ensemble-learning method by integrating SVM, the decision tree and KNN algorithm into our predictive model based on the selected features for intracerebral haemorrhage prediction. Finally, we trained and tested the model by optimizing the key parameters and validating the model predictive capacity, respectively.

#### Sample size estimation

Limited by resources and ethical reasons, it is impossible to obtain an infinite sample size for the optimization of the key parameters of the model, so Eq. 1 computes the optimum sample size (n) to meet statistical significance [27].

1.1 $$ n={\left[\frac{2\left({u}_{\alpha }+{u}_{\beta}\right)\sigma }{\delta}\right]}^2 $$

1.2 $$ d=\frac{\mu_1\hbox{-} {\mu}_2}{\sigma }=\frac{\delta }{\sigma } $$

1.3 $$ n={\left[\frac{2\left({u}_{\alpha }+{u}_{\beta}\right)}{d}\right]}^2 $$

Eq. 1.1 denotes σ as the standard deviation; *u*_*α*_ and *u*_*β*_ as the critical values of the u-test at the first type of error rate and the second type of error rate, respectively.

Eq. 1.2 defines effect size d [26] as *δ*/*σ*; *μ*_1_ and *μ*_2_ as the mean values of the disease and the control data, respectively. The first and the second type of error *α* and *β* were set to 0.05 and 0.1, respectively. Indicated by Kabacoff et al. [26], δ represents *μ*_1_ ‐ *μ*_2_. Then, we re-wrote Eq. 1.1 as Eq. 1.3.

#### Feature selection

After locating the sample size, we employed a (Additional file 1: Figure S1) statistical significance test for these candidate features, and the digital values of this test were extracted by using RadidaDicomViewer software [26].

#### Machine learning methods

In the beginning, this study employed three commonly used classification algorithms [17–19] such as KNN, decision tree and SVM to develop the predictive model. Then, we integrated these three classic methods detailed in the supplemental methods section into a novel ensemble learning [16] model to improve our predictive accuracy. Here, we employed Matlab2014a [28] to implement these methods with the default parameter setup and the parameter k of KNN was set to 5. We describe the ensemble-learning algorithm (Additional file 1: Figure S2) as follows.

**Input:** Sample set S = {(*x*_1_, *y*_1_), (*x*_2_, *y*_2_), ⋯, (*x*_*n*_, *y*_*n*_)}, where *x*_*n*_ is the example and *y*_*n*_ ∈ {0, 1} is the label; weak classifier $\mathcal{L}\in \left\{{\mathcal{L}}_1=\mathrm{SVM},{\mathcal{L}}_2=\mathrm{Decision}\ \mathrm{tree},{\mathcal{L}}_3=\mathrm{KNN}\right\}$. T is the iteration number.

**Process:**

1. for m = 1,…, $$ \mathcal{L} $$
2. Initialize the weight distribution $$ {D}_1(i)=\raisebox{1ex}{$1$}\!\left/ \!\raisebox{-1ex}{$n$}\right. $$; (n is the number of examples and i is the index of the example)
3. for t = 1,…,T
4. Based on the sample distribution Dt and ${\mathcal{L}}_m$, we train the weak classifier *h*_*t*_
5. Compute the error (*ε*_*t*_) for *h*_*t*_
  2 $$ {\varepsilon}_t=\frac{number\ of\ incorrectly\ classified\ examples}{total\ number\ of\ examples} $$
6. Compute the weight (*α*_*t*_) for *h*_*t*_
  3 $$ {\alpha}_t=\frac{1}{2}\mathit{\ln}\frac{1-{\varepsilon}_t}{\varepsilon_t} $$
7. Update the weight for each sample
  4 $$ {D}_{t+1}\left(\mathrm{i}\right)=\frac{D_t(i)}{sum(D)}\left\{\begin{array}{c}\exp \left(-{\alpha}_t\right)\  if{h}_t\left({x}_i\right)={y}_i\\ {}\exp \left({\alpha}_t\right)\  if{h}_t\left({x}_i\right)\ne {y}_i\end{array}\right. $$
8. End
9. Obtain the ensemble learning classifier *H*_*m*_ by the adaboost algorithm [29, 30]
  5 $$ {H}_m(x)=\operatorname{sign}\left(f(x)\right)=\mathit{\operatorname{sign}}{\sum}_{t=1}^T{\alpha}_t{h}_t(x) $$
10. Calculate the accuracy of *H*_*m*_
  6 $$ {P}_{H_m}=\frac{\mathrm{number}\  of\ correctly\ classified\ examples}{total\ number\ of\ examples} $$
11. End
12. Assign a weight ${\mathrm{w}}_{{\mathrm{H}}_{\mathrm{m}}}$ to each H_m_
  7 $$ {w}_{H_m}=\frac{1}{2}\mathit{\ln}\frac{P_{H_m}}{1-{P}_{H_m}} $$

**Output:** anomaly ensemble

8 $$ Y(x)=\operatorname{sign}{\sum}_{m=1}^3{w}_{H_m}{H}_m(x) $$

## Results

### Sample size estimation

We used Eq. 1 to compute the sample size by setting parameter d [26]. The sample size of the lower bound was 66. Half the samples were the control and the rest were the disease.

### Statistical test results for the candidate features

Since there were ten potential features closely related to the intracerebral haemorrhage [2, 4, 9, 12, 31, 32], this study chose them as the candidate features (Additional file 1: Table S5) to develop the predictive model. Then, we employed the statistical test workflow [33] (Additional file 1: Figure S1) to verify the statistical significance of each potential feature.

**Fig. 3:**
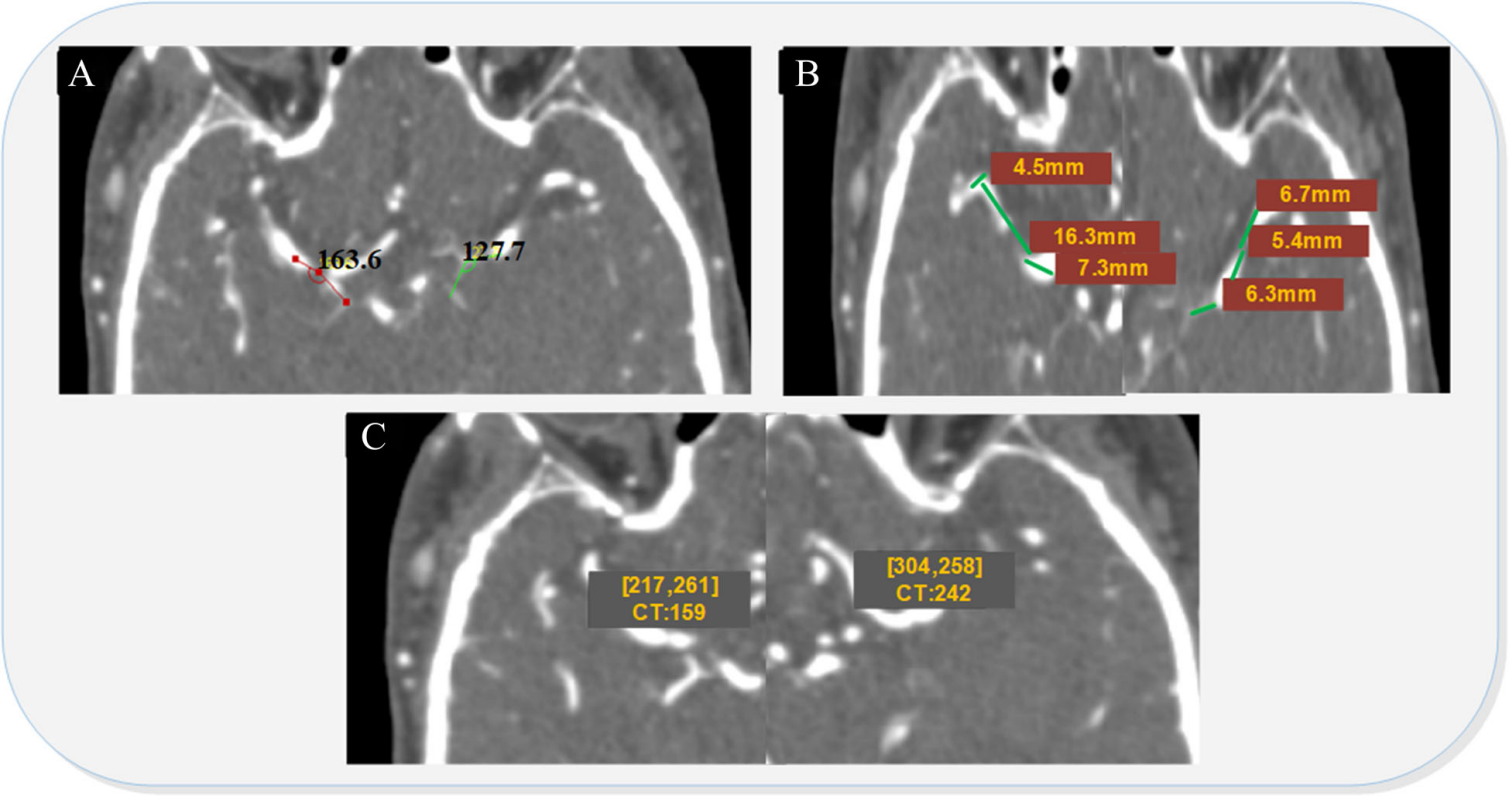
Computed tomography angiography features for the haematoma side and contralateral side of ICH patients

Additional file 1: Table S5 demonstrates that the angle between the middle cerebral artery and artery (AMIC), the distance between the beginning of the median cerebral artery and the superior trunk (DMS) and the CT value of the lenticulostriate artery (CTL) were statistically significant between the control and the disease data set since they have small *p* values. To confirm these statistical results, we collected a clinical CT image to investigate these three potential features. Figure 3 shows that the angle of the AMIC was 127.7 degrees, the length of the DMS was 18.4 mm and the CTL was 242 HU on the haematoma side, whereas the angle of the AMIC was 163.6 degrees, the length of the DMS was 28.1 mm and CTL was 158 HU in the non-haematoma side. It is obvious that the angle of the AMIC on the haematoma side was smaller than the angle of the AMIC on the non-haematoma side, the length of DMS on the haematoma side was much shorter than the length of DMS on the non-haematoma side, and the CTL on the haematoma side was greater than the CTL on the non-haematoma side.

### Predictive performance for each model

We employed SVM, decision tree, KNN and ensemble learning methods to develop the predictive model by using AMIC, DMS and CTL as the features.

**Fig. 4:**
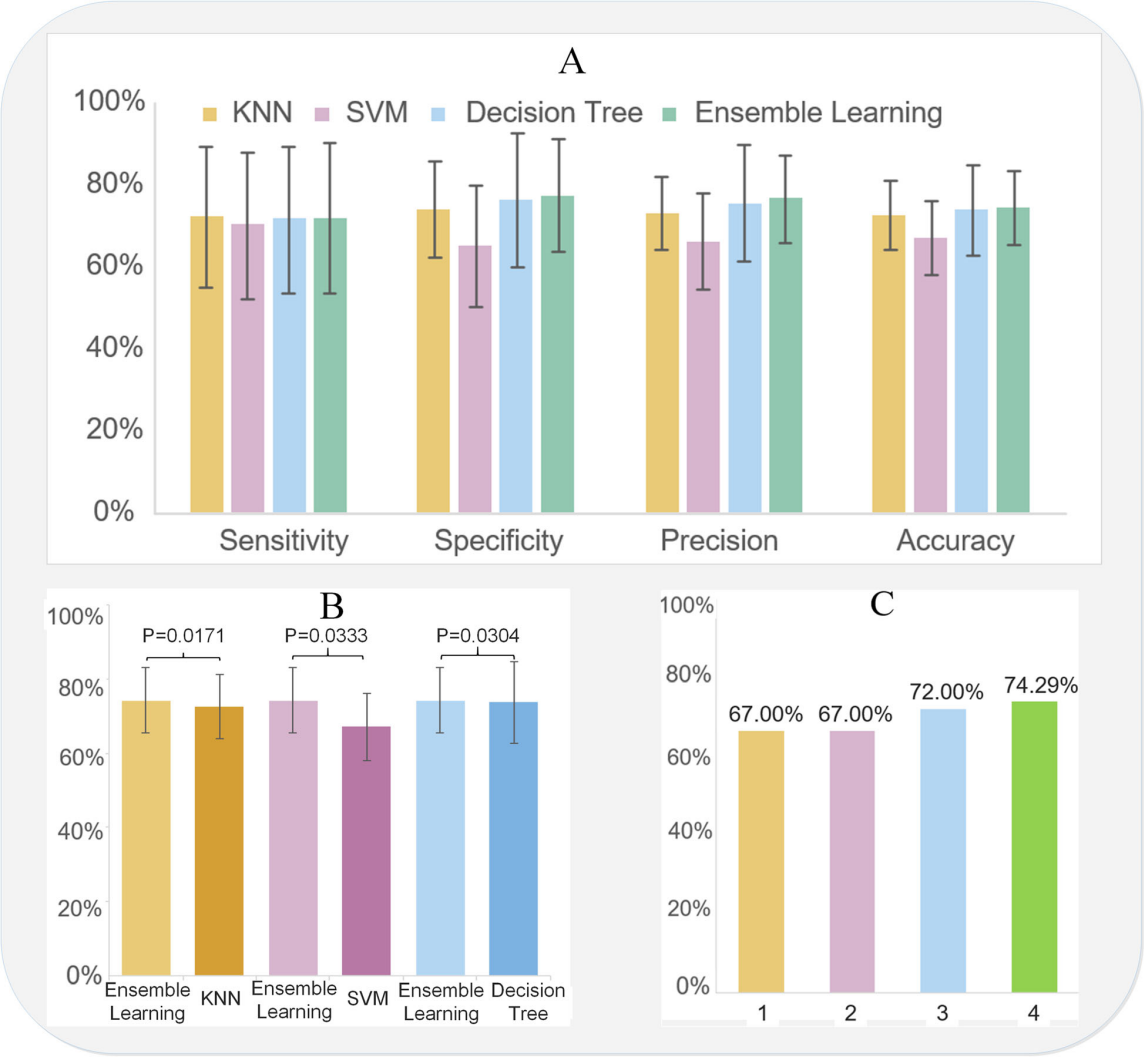
Model performance. **a**. General model performance; **b**. Comparison among ensemble learning, KNN, decision tree and SVM; **c**. Comparison of previous similar research (Index 1, 2, 3 and 4 indicate the references [20–22] and our model, respectively)

Here, we employed cross validation [34] to train and test each model. We divided the data set into eight groups, 6 training sets and 2 test sets, to train and test the data. Figure 4a and Additional file 1: Table S7 show the sensitivity, specificity, precision and accuracy for KNN, SVM, decision tree and ensemble learning, in which ensemble learning outperforms the other three. Additional file 1: Table S6 details four classic classification measurement standards (sensitivity, specificity, precision and accuracy).

Moreover, since the ensemble-learning algorithm employs the accuracy measurement as the objective function to optimize the key weight of the weak classifiers, here, we compare the predictive capacity among ensemble learning, SVM, KNN and decision tree in Fig. 4b and compare the predictive capacity between ensemble-learning and previous research [20–22] in Fig. 4c. Figure 4b and c demonstrate that ensemble learning performs best in the prediction of intracerebral haemorrhage based on the accuracy measurements with statistically significant results.

## Discussion

Because of the high morbidity and mortality of intracerebral haemorrhage, the prevention and treatment of intracerebral haemorrhage is currently an issue of great concern. Research on the prediction of intracerebral haemorrhage is becoming increasingly important. Currently, the relative risk between intracerebral haemorrhage and hypertension, diabetes, hyperlipemia, or other systemic diseases is widely accepted, but these diseases correlate to other cardio-cerebral vascular incidents, such as ischaemic stroke and coronary heart disease, with poor specificity [15, 35]. In clinical practice, it is still difficult to predict the direct haemorrhagic risk for these high-risk populations. There are many studies [15, 35] on the prediction of haematoma expansion after intracerebral haemorrhage by using CT images of the brain, such as spot signs. However, no other study has identified the haemorrhagic risk for the non-intracerebral haemorrhage potential patients, which may be the reason for the negative results of many intracerebral haemorrhage clinical trials by mixing these subpopulations with others. If so, risk identification might greatly facilitate precise control and prevention of the increasing occurrences of intracerebral haemorrhage [3].

ICH in the basal ganglion, which constitutes the majority of ICH subtypes that are frequently related to hypertensive vasculopathy, often occurs due to the rupture of small vessels, especially the branches of lenticulostriate artery, releasing the blood into the brain parenchyma [2]. The lenticulostriate artery usually arises from the trunk of the middle cerebral artery before the bifurcation. The intracerebral segments of the lenticulostriate artery with its branches are shaped as a curve or a loop, which results in much more pressure on the vessel wall when it flows through the bending portion. Therefore, the branches of the lenticulostriate artery bear much more shear stress or pressure due to the decreasing diameter and the unique morphology of the lenticulostriate artery and its branches, which becomes a high-risk factor for intracerebral haemorrhage [36, 37]. For this reason, the present study chose ten potential characteristics (Additional file 1: Table S3) associated with the shear stress of the lenticulostriate artery on computed tomography angiography imaging.

The present study found that the angle between the middle cerebral artery and the internal carotid artery (AMIC), the distance between the beginning of the median artery and superior trunk (DMS), and the CT of the lenticulostriate artery (CTL) are statistically significant enough to be causes of intracerebral haemorrhage (Additional file 1: Table S5). In addition, we employed these causes as the features of the classification model to predict the occurrence of intracerebral haemorrhage (Fig. 2). As we know, shear stress arises from the friction between blood flow and the vascular endothelium, paralleling the vessel wall. The magnitude of shear stress in straight vessels is directly proportional to the viscosity of blood and blood flow and inversely proportional to the third power of the inner radius of the vessel. The three characteristics we chose as features in the present study may be closely associated with the local shear stress around the basal ganglion, especially the density (CT value) of the lenticulostriate artery, as the greatest change in blood pressure and velocity of blood flow occurs at the transition from arterioles to capillaries. Briefly, the narrow AMIC (Fig. 3a) and short DMS (Fig. 3b) may lead to high impact forces towards the branches of the middle cerebral artery, while the high CT value in lenticulostriate arterial areas (CTL) illustrates tighter arterioles with possible weak vessel walls that receive the shear stress of the blood stream (Fig. 3c).

Moreover, since accuracy is an important measurement for clinical personnel [21] and the function of our ensemble-learning model, we compared the performance of the ensemble-learning method with previous similar studies and our three methods (KNN, decision tree and SVM), as shown in Fig. 4b and c, respectively. Figure 4b and c demonstrate that these features are so efficient that our developed ensemble-learning method outperforms not only previous work [20–22] but also the other three methods based on accuracy measurements with statistically significant results. However, Fig. 4a shows that the sensitivity of ensemble learning was not better than that of the KNN method. Since the ensemble learning method employs accuracy as the objective function to optimize the key weights (Eq. 3 and Eq. 4) for each weak classifier, it cannot guarantee the best performance for the other three measurements, including sensitivity.

While this study demonstrated better performance in stroke prediction, it still has several drawbacks. For example, our sample size was too small for us to consider more classification measurements for the predictive model. Therefore, we are going to integrate more recent bioinformatics research algorithms [38–43] into this system and employ a large CTA imaging data set to overcome the current shortcomings.

In conclusion, AMIC, DMS and CTL can predict intracerebral haemorrhage. Our developed ensemble-learning method efficiently employs these selected features to efficiently predict the probability of intracerebral haemorrhage.

## Conclusions

In this article, we propose a mathematical framework for the prediction of intracerebral haemorrhage. Statistical methods are employed to compute the size of the samples and demonstrate which candidate biomarkers are statistically significant enough to be the features of the model. A novel ensemble-learning method is developed by integrating SVM, decision tree and KNN algorithms into the model to improve the predictive accuracy. Our research results reveal that AMIC, DMS and CTL can predict intracerebral haemorrhage, and the developed mathematical model in the present study not only outperforms the classic KNN, decision tree and SVM models but also performs better than previous similar research.

## Additional file

Additional file 1: Basic information of 151 intracerebral haemorrhage patients in the present study; Supplementary tables and figures. **Table S1.** Distribution of age group in patients with cerebral. **Table S2.** The complications of patients with intracerebral haemorrhage. **Table S3.** The candidate features. **Table S4.** The digital values of the candidate feature from the CT imaging samples. **Table S5.** *P* value of the statistical test for each candidate feature. **Table S6.** The standard of the classification measurement. **Table S7.** The classification results. **Figure S1.** Work flow of the statistical test. **Figure S2.** Workflow of the ensemble learning. (PDF 130 kb)

## Abbreviations

KNN: K-Nearest Neighbour
SVM: Support Vector Machine
