# Supplementary material for: Computed tomography angiography-based analysis of high-risk intracerebral haemorrhage patients by employing a mathematical model
Source: BMC Bioinformatics. 2019 May 1;20(Suppl 7):193. doi: 10.1186/s12859-019-2741-5 (PMC6509873; doi:10.1186/s12859-019-2741-5)
Supplement: Supplementary file 1 — Basic information of 151 intracerebral haemorrhage patients in the present study; Supplementary tables and figures. Table S1. Distribution of age group in patients with cerebral. Table S2. The complications of patients with intracerebral haemorrhage. Table S3. The candidate features. Table S4. The digital values of the candidate feature from the CT imaging samples. Table S5. P value of the statistical test for each candidate feature. Table S6. The standard of the classification measurement. Table S7. The classification results. Figure S1. Work flow of the statistical test. Figure S2. Workflow of the ensemble learning. (PDF 130 kb) [file 12859_2019_2741_MOESM1_ESM.pdf]

The ordinary differential equations of AA metabolic network

December 29, 2018

Construction of the AA metabolic network model in human PMN

Method

On the basis of the AA metabolic network, a set of ODEs were constructed to describe cell behavior of inflammation in human PMN (see details in Supplementary information). The ode 45 s routine of Matlab was used to integrate the ODEs. Michaelis Menten equations (equation (3)) were used to describe enzyme catalytic reactions in the network:

$$\frac{d[S]}{dt} = \frac{K_{cat}[E_t][S]}{K_m + [S]} \tag{1}$$

where $[S]$ is the concentration of the substrate, $[E_t]$ is the total concentration of enzyme, K_{cat} is turnover number, and K_m is the Michaelis-Menten constant.
If competitive reversible inhibitors are involved in the catalysis, the equation is:

$$\frac{d[S]}{dt} = \frac{K_{cat}[E_t][S]}{K_m(1 + \frac{[I]}{K_i}) + [S]} \tag{2}$$

where $[I]$ is the concentration of inhibitor and K_i is the inhibition constant, which is defined as:

$$K_i = \frac{[E][I]}{[EI]} \tag{3}$$

If the inhibitors are irreversible, we assume the enzymes would decay according to the following equation:

$$\frac{d[E]}{dt} = -K[E][I] \tag{4}$$

where K is a constant.
When activators are involved in the catalysis, we use the following equation:

$$\frac{d[S]}{dt} = \frac{K_{cat}(1 + ([A]/KI))[E_t][S]}{K_m + [S]} \tag{5}$$

where $[A]$ is the concentration of activator and KI is a constant.
When up regulation occurred though transcription, we described its effect with the following equation:

$$\frac{d[E]}{dt} = \frac{k[g]^2}{[g]^2 + [k]^2} \tag{6}$$

where $[g]$ is the concentration of the metabolite up regulating the transcription of the enzyme, K and k are constants.

Equations

The ordinary differential equations (ODEs)of the AA metabolic network was developed. A series of ODEs was established to simulate unicellular behavior, which included 24 initial concentrations and 45 reaction constants (as the following equations). The ODEs for PMNs are:

$$(1)\ \frac{d[AA]}{dt} = \frac{K_{cat,PLA2}(1+\frac{[12-HPETE]}{K_{12HPETE\to PLA2}}+\frac{[15-HPETE]}{K_{15HPETE\to PLA2}}+\frac{[LTB4]}{K_{LTB4\to PLA2}}+\frac{[5-HETE]}{K_{5HETE\to PLA2}})[PLA2][PL]}{K_{m,PLA2}(1+\frac{[AA]}{K_i})+[PL]}$$

$$-\frac{K_{cat,15LOX}[15-LOX][AA]}{K_{m,15LOX}(1+\frac{[15-HPETE]}{K_i})+[AA]}-\frac{K_{cat,12LOX}[12-LOX][AA]}{K_{m,12LOX}(1+\frac{[12-HPETE]}{Ki_{12HPETE\to 12LOX}}+\frac{[15-HETE]}{Ki_{15HPETE\to 12LOX}})+[AA]}$$

$$-\frac{K_{cat,5LOX}[5-LOX][AA]}{K_{m,5LOX}(1+\frac{[5-HPETE]}{K_i}+\frac{[12-HETE]}{Ki_{12HETE\to 5LOX}}+\frac{[15-HETE]}{Ki_{15HETE\to 5LOX}}+\frac{[PGE2]}{Ki_{PGE2\to 5LOX}}+\frac{[5-HETE]}{Ki_{5HETE\to 5LOX}})+[AA])}$$

$$-\frac{K_{cat,COX2}[COX-2][AA]}{K_{m,COX2}(1+\frac{[PGH2]}{K_i}+\frac{[PGE2]}{Ki_{PGE2\to COX2}})+[AA]}-Kd_{exoAA}[exoAA]-Kd_{AA}[AA]$$

$$(2)\ \frac{d[15-HPETE]}{dt}=\frac{K_{cat,15-LOX}[15-LOX][AA]}{K_{m,15LOX}(1+\frac{[15-HPETE]}{K_i})+[AA]}-\frac{K_{cat,PHGPx}[PHGPx][15-HPETE]}{K_{m,PHGPx}(1+\frac{[15-HETE]}{K_i})+[15-HPETE]}-Kd_{15HPETE}[15-HPETE]$$

$$(3)\ \frac{d[15-HETE]}{dt}=\frac{K_{cat,PHGPx}[PHGPx][15-HPETE]}{K_{m,PHGPx}(1+\frac{[15-HETE]}{K_i})+[15-HPETE]}-Kd_{15-HETE}[15-HETE]$$

$$(4)\ \frac{d[12-HPETE]}{dt}=\frac{K_{cat,12-LOX}[12-LOX][AA]}{K_{m,12LOX}(1+\frac{[12-HPETE]}{Ki_{12-HPETE\to 12-LOX}}+\frac{[15-HETE]}{Ki_{15-HPETE\to 12-LOX}}+[AA]}-\frac{K_{cat,PHGPx}[PHGPx][12-HPETE]}{K_{m,PHGPx}(1+\frac{[12-HETE]}{K_i})+[12-HPETE]}$$

$$(5)\ \frac{d[12-HETE]}{dt}=\frac{K_{cat,PHGPx}[PHGPx][12-HPETE]}{K_{m,PHGPx}(1+\frac{[12-HETE]}{K_i})+[12-HPETE]}$$

$$(6)\ \frac{d[PGH2]}{dt}=\frac{K_{cat,COX2}[COX-2][AA]}{K_{m,COX2}(1+\frac{[PGH2]}{K_i}+\frac{[PGE2]}{Ki_{PGE2\to COX2}})+[AA]}-\frac{K_{cat,TXAS}[TXAS][PGH2]}{K_{m,TXAS}(1+\frac{[TXA2]}{K_i})+[PGH2]}$$

$$-\frac{K_{cat,PGES}[PGES][PGH2]}{K_{m,PGES}(1+\frac{[PGE2]}{K_i}+\frac{[AA]}{Ki_{AA\to PGES}}+\frac{[15-HETE]}{Ki_{15-HETE\to PGES}})+[PGH2]}$$

$$(7)\ \frac{d[PGE2]}{dt}=\frac{K_{cat,PGES}[PGES][PGH2]}{K_{m,PGES}(1+\frac{[PGE2]}{K_i}+\frac{[AA]}{Ki_{AA\to PGES}}+\frac{[15-HETE]}{Ki_{15-HETE\to PGES}})+[PGH2]}$$

$$(8)\ \frac{d[TXA2]}{dt}=\frac{K_{cat,TXAS}[TXAS][PGH2]}{K_{m,TXAS}(1+\frac{[TXA2]}{K_i})+[PGH2]}-Kd_{TXA2}[TXA2]$$

$$(9)\ \frac{d[TXB2]}{dt}=Kd_{TXA2}[TXA2]-Kd_{TXB2}[TXB2]$$

$$(10)\ \frac{d[5-HPETE]}{dt}=\frac{K_{cat,5-LOX}[5-LOX][AA]}{K_{m,5-LOX}(1+\frac{[5-HPETE]}{K_i}+\frac{[12-HETE]}{Ki_{12-HETE\to 5-LOX}}+\frac{[15-HETE]}{Ki_{15-HETE\to 5-LOX}}+\frac{[PGE2]}{Ki_{PGE2\to 5LOX}}+\frac{[5-HETE]}{Ki_{5-HETE\to 5-LOX}})+[AA]}$$

$$-\frac{K_{cat,5-LOX}[5-LOX][5-HPETE]}{K_{m,5-LOX}(1+\frac{[LTA4]}{K_i}+\frac{[12-HETE]}{Ki_{12-HETE\to 5-LOX}}+\frac{[15-HETE]}{Ki_{15-HETE\to 5-LOX}}+\frac{[PGE2]}{Ki_{PGE2\to 5-LOX}}+\frac{[5-HETE]}{Ki_{5-HETE\to 5-LOX}})+[5-HPETE]}$$

$$-\frac{K_{cat,PHGPx}[PHGPx][5-HPETE]}{K_{m,PHGPx}(1+\frac{[5-HETE]}{K_i})+[5-HPETE]}$$

(11) $$\frac{d[5-HETE]}{dt} = \frac{K_{cat,PHGPx}[PHGPx][5-HPETE]}{K_{m,PHGPx}(1+\frac{[5-HETE]}{K_i}) + [5-HPETE]} - Kd_{5HETE}[5-HETE]$$

(12) $$\frac{d[LTA4]}{dt} = \frac{K_{cat,5LOX}[5-LOX][5-HPETE]}{K_{m,5-LOX}(1+\frac{[LTA4]}{K_i} + \frac{[12-HETE]}{Ki_{12-HETE\to 5-LOX}} + \frac{[15-HETE]}{Ki_{15-HETE\to 5-LOX}} + \frac{[PGE2]}{Ki_{PGE2\to 5-LOX}} + \frac{[5-HETE]}{Ki_{5-HETE\to 5-LOX}}) + [5-HPETE]}$$
$$- \frac{K_{cat,LTA4H}[LTA4H][LTA4]}{K_{m,LTA4H}(1+\frac{[LTB4]}{K_i}) + [LTA4]} - Kd_{LTA4}[LTA4]$$

(13) $$\frac{d[LTB4]}{dt} = \frac{K_{cat,LTA4H}[LTA4H][LTA4]}{K_{m,LTA4H}(1+\frac{[LTB4]}{K_i}) + [LTA4]} - Kd_{LTB2}[LTB2]$$
$$- \frac{K_{cat,CYP4F3}[CYP4F3][LTB4]}{K_{m,CYP4F3}(1+\frac{[20-OH-LTB4]}{K_i} + \frac{[12-HETE]}{Ki_{12-HETE\to CYP4F3}} + \frac{[5-HETE]}{Ki_{15-HETE\to CYP4F3}}) + [LTB4]} - Kd_{LTB4}[LTB4]$$

(14) $$\frac{d[20-OH-LTB4]}{dt} = \frac{K_{cat,CYP4F3}[CYP4F3][LTB4]}{K_{m,CYP4F3}(1+\frac{[20-OH-LTB4]}{K_i} + \frac{[12-HETE]}{Ki_{12-HETE\to CYP4F3}} + \frac{[5-HETE]}{Ki_{5-HETE\to CYP4F3}}) + [LTB4]}$$

(15) $$\frac{d[PLA2]}{dt} = 0$$

(16) $$\frac{d[15-LOX]}{dt} = \frac{k_{PGE2\to 15-LOX}[PGE2]^2}{[PGE2]^2 + (K_{PGE2\to 15-LOX})^2} - Kd_{15-LOX}[15-LOX]$$

(17) $$\frac{[12-LOX]}{dt} = -Ki_{15-HPETE\to 12-LOX}[15-HPETE][12-LOX]$$

(18) $$\frac{d[COX-2]}{dt} = 0$$

(19) $$\frac{d[PGES]}{dt} = 0$$

(20) $$\frac{d[TXAS]}{dt} = -(Ki_{15-HPETE\to TXAS}[15-HPETE] + Ki_{PGH2\to TXAS}[PGH2])[TXAS]$$

(21) $$\frac{d[5-LOX]}{dt} = (K_{LTB4\to 5-LOX}[LTB4] - Ki_{LTA4\to 5-LOX}[LTA4] - Ki_{5-HPETE\to 5-LOX}[5-HPETE]$$
$$- Ki_{15-HPETE\to 5-LOX}[15-HPETE])[5-LOX]$$

(22) $$\frac{d[LTA4H]}{dt} = -\frac{K_{cat,LTA4H}[LTA4H][LTA4]}{129(K_{m,LTA4H} + [LTA4])}$$

(23) $$\frac{d[CYP4F3]}{dt} = 0$$

(24) $$\frac{d[PHGPx]}{dt} = 0$$
